# Effect of framework type on survival probability of implant-supported temporary crowns: An *in vitro* study

**DOI:** 10.4317/jced.56292

**Published:** 2020-05-01

**Authors:** Vinícius-Anéas Rodrigues, Amanda-Maria-de Oliveira Dal Piva, Claudio-Akira Yamaguchi, Alexandre-Luiz-Souto Borges, Marcio-Katsuyoshi Mukai, João-Paulo-Mendes Tribst

**Affiliations:** 1 DDs, MSc, PhD, Department of Dental Materials and Proshodontics, Faculty of Pindamon­hangaba (FUNVIC), Pindamonhangaba/SP, Brazil; 2 DDs, MSc, PhD, Department of Dental Materials and Proshodontics, São Paulo State University (Unesp), Institute of Science and Technology, São José dos Campos / SP, Brazil; 3 DDs, MSc at Department of Prosthodontics, School of Dentistry, University of Sao Paulo, Sao Paulo, SP, Brazil

## Abstract

**Background:**

This *in vitro* study evaluated the effect of framework type on the survival probability of temporary implant-supported crowns and on the implant platform structure after dynamic fatigue.

**Material and Methods:**

Thirty (30) external hexagon implants (3.75 x 10 mm) were embedded in acrylic resin following the ISO-14801. Standardized temporary crowns (n=10, N=30) were manufactured in acrylic resin and divided according to the framework type: Total plastic, Plastic with CoCr base and Titanium. The crowns were installed onto the implants (20N.cm) and fatigued (100N, 2 Hz) to determine the crowns’ survival probability for missions of 300.000 and 600.000 cycles. Fatigue data were submitted to the Kaplan-Meier test followed by Wilcoxon and Log Rank, all with α = 5%. The implant platforms were parametrically inspected based on the scanning before and after the fatigue to evaluate the damage. The strain values were analyzed using One-way ANOVA and Tukey test, all with α = 5%.

**Results:**

ANOVA revealed that the Total plastic showed less implant damage (-0.07 ± -0.03 mm) than the Plastic with CoCr base (-0.08 ± -0.04 mm) and the Titanium (-0.10 ± -0.01 mm) frameworks. Therefore, the framework type to manufacture implant-supported temporary crowns influences the fatigue survival of the restoration and the implant platform damage. The Plastic with CoCr base and Titanium frameworks showed superior reliability than the Total plastic framework which could not survive 600,000 cycles.

**Conclusions:**

The Plastic with CoCr base and the Titanium framework are suitable for restorations over 3 months in use, without a difference in the implant platform damage.

** Key words:**Implant dentistry, axial loading, occlusion, methodo­logical study.

## Introduction

Manufacturing a provisional crown immediately after implant placement promotes esthetics, assists in obtaining a favorable emergence profile and serves as a model for the final restoration (1-3). Among the dental materials available for use as provisional restorations, Poly(methyl methacrylate) (PMMA) is widely reported as a suitable material due to its practicality and adequate mechanical properties (4,5). For unitary implants, PMMA provisional crowns are usually associated with the use of a metallic framework (6-8). However, it is possible to find reports of totally metallic (Titanium cylinder) (9), totally plastic or plastic with a metal base (plastic cylinder with Co-Cr) implant/crown connections (10,11).

A plastic cylinder is usually indicated for the casting process of the final crown, but may present an elevated vertical misfit (10-12). Therefore, the possibility of using plastic framework for provisional crowns may be favorable because of its reduced cost, shorter preparation time and ease for performing an intraoral adjustment (1,11). Previous case reports affirm that the plastic cylinder was used as a provisional framework to achieve improved esthetic results due to the absence of metal1 (13-15). However, whether this framework is able to resist masticatory forces has not yet been investigated to justify its clinical use.

Since the removal of a temporary crown is not suiTable to preserve the shape of the soft tissue around the implant, the movement of the provisional restoration may also have a deleterious effect on the potential augmentation and healing of the surgical site (16). The provisional crown should be stable on the implant and kept in position without removal for 3 months when placed on an immediate implant surgery (16). For this reason, if a plastic framework is selected for manufacturing a provisional crown, it should be strong enough to avoid mechanical problems. There is currently no available scientific information which shows the performance on dynamic fatigue of different frameworks for implant-supported temporary crowns (11). Thus, the purpose of this study was to evaluate the effect of framework type on the survival probability of temporary implant-supported crowns and on the implant platform structure after dynamic fatigue. The null hypothesis was that there would be no influence of framework type on the survival probability of temporary implant-supported crowns.

## Material and Methods

-Sample preparation

For this study, thirty (30) hexagon extern implants (3.75 x 10 mm, Conexão Sistemas de Prótese, São Paulo, SP, Brazil) were embedded into polyurethane resin (F160-resin Axson Brasil Industria e Comercio Ltda), keeping 3mm of threads out of the resin (17). To do so, the implants were fixed in a parallelometer apparatus (Bio-Art 2, Bio-Art Equipamentos Odontológicos) to maintain the correct position of the implants perpendicular to the ground plane until complete polymerization of the resin. The implants were randomly distributed into 3 groups according to the framework type used for temporary crown manufacture: Plastic, Plastic with CoCr base and Titanium (Conexão Sistemas de Prótese, São Paulo, SP, Brasil). All frameworks had the same external geometry with superficial grooves. Each cylinder then received a uniform layer of acrylic resin on its surface by brushing to ensure intimate contact between the PMMA and the framework’s surface ( Table 1).

**Table 1:**
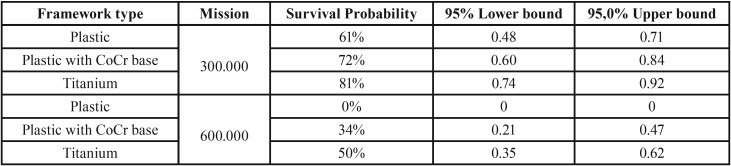
Survival probability for temporary implant supported crowns after missions of 300.000 and 600.000 cycles, according to the framework type.

A wax patter with standardized anatomy of a first lower right molar was made to manufacture a temporary crown in acrylic resin (Duralay, Reliance Dental Mfg Co, Worth, Illinois). Next, an impression was made using a combination of putty and light-body vinyl polysiloxane (VPS) material (Elite HD, Zhermack, Italy). This VPS matrix was subsequently sectioned and used to fabricate 29 more crowns used in the dynamic fatigue test. The crowns were perforated (3.8 mm in diameter) with the aid of a tungsten carbide drill and placed on each framework already screwed onto the implant. The crown was fixed with self-curing acrylic resin with the aid of a fine-tipped brush on the implant. Then, the cylinder was cut at occlusal height and the provisional crowns were polished with abrasive tips (Edenta AG, Au/SG, Switzerland). Each crown was installed with a manual torque wrench of 20N.cm.

-Dynamic Fatigue test

The fatigue loading test was run in water at a temperature of 37°C with 100 N at 2 Hz. The load was applied using a stainless-steel round tip (6 mm diameter), which was centrally positioned at the occlusal surface of the crown at the central fossa (18). The dynamic fatigue test was carried out until all samples catastrophically failed, defined as the crown or screw fracture. All specimens were examined (7.5× magnification) at the end of each 100.000 cycles under optical light microscopy (Leica MZ 125; Leica Microsystems GmbH, Wetzlar, Germany) and the survival samples returned to the fatigue test in the same position. In order to ensure the same position during the entire test, each sample received two marks with diamond bur in the laterals of the fixation cylinder.

-Parametric inspection of external hexagon 

After the fatigue test, each implant was scanned until the first thread (CEREC AC Omnicam, Sirona, São Paulo, SP, Brazil) and the stereolithography file (STL) was stored in a CAD system (Rhinoceros 5.0 SR9, McNeil). The 3D STL files of the fatigued samples facets were subsequently collected and superimposed from a non-fatigued implant baseline (Converted in STP) using a 3D digital parametric inspection software (GOM Inspect, Braunschweig, Germany) with alignment in a local coordinate system. The highest values were manually selected using point cloud inspection and following the strain colorimetric scale, and then submitted to statistical analysis (19).

-Data analysis

For the fatigue data, Minitab statistical software (Version 14.12, 2004) was used to compare the survival probability of the groups using the Kaplan-Meier survival curve. The influence of the framework was observed by comparing the survival curves using the Log-rank and Wilcoxon tests at a 0.05 significance level. A difference between the groups was assumed if there were non-overlapping confidence intervals between them. Missions of 300.000 and 600.000 cycles were selected for the survival probability, simulating approximately 3 and 6 months of use in the mouth (20). The data were analyzed by one-way ANOVA followed by post-hoc Tukey test for the parametric inspection, all with α=5%.

## Results

**Figure 1:**
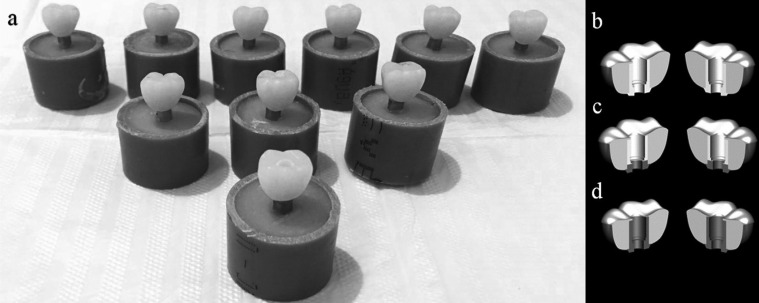
(a-d) a) Samples embedded into resin for the fatigue test, b) Schematic illustration of the plastic framework, c) Schematic illustration of the plastic framework with CoCr base and d) Titanium framework.

Due to the low *p*-value for both Wilcoxon (Chi-Squared = 28.85, *P* < 0.001) and Log Rank (Chi-Squared = 42.98, *P* < 0.001) tests, it was possible to assume that there is a significant difference between survival curves. The survival difference should be observed by interposing the confidence intervals for each group. There was no significant difference between the use of Plastic framework with CoCr and the Titanium or Plastic for an initial mission of 300.000 cycles. However, Titanium showed higher reliability compared to the Plastic framework. Meanwhile, only the Plastic framework with CoCr and the Titanium framework survived for a mission of 600.000 cycles, without any difference between them (Fig. 1).

**Figure 2:**
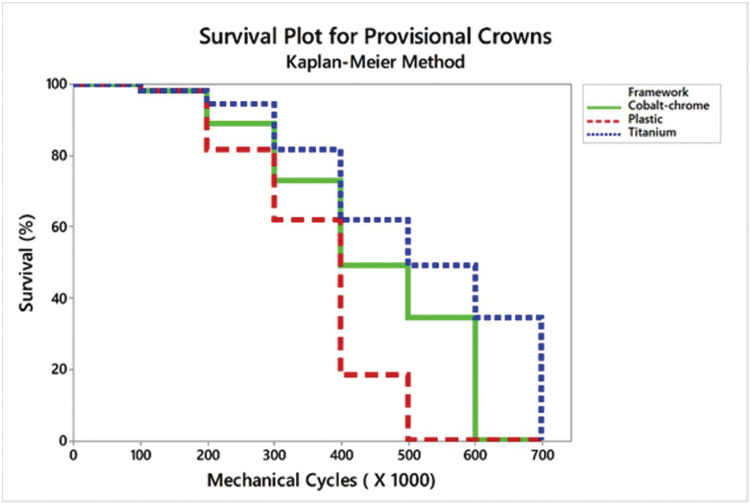
Kaplan-Meier survival plot for each group.

The microscopy analysis (7.5x magnification) showed a qualitative difference in the damage pattern generated on the prosthetic platform in the external hexagon according to the framework type (Fig. 2).

The implants were scanned before and after the dynamic fatigue test and parametrically inspected in 3D analysis software (GOM Inspect, Braunschweig, Germany) for the quantitative damage analysis to the implant platform of the external hexagon. The highest damage occurred at the edges of the external hexagon. The deformation peak was measured in each sample to obtain the mean deformation value and standard deviation per group ( Table 2). One-way ANOVA showed that there was a difference between the groups (*p* = 0.043).

**Table 2:**
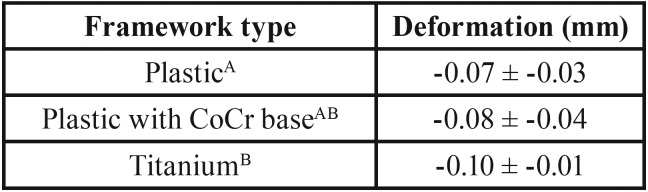
Descriptive statistical analysis (mean deformation value and standard deviation) and Tukey test grouping, according to the framework type.

## Discussion

This study aimed to evaluate the effect of different types of framework on the survival probability of implant-supported temporary crowns. The null hypothesis was rejected since the crowns reliability was significantly influenced by the framework type. The literature reports that a prefabricated plastic abutment can be used as a temporary framework because it provides adequate fitting, easy customization, and promotes a more esthetic crown (1). However, the results of the present study also demonstrated that the crown onto a plastic framework presented low survival probability during fatigue when compared to plastic framework with CoCr base or a titanium framework. Two different cycling missions were evaluated: 300.000 and 600.000 cycles, which are equivalent to approximately three and six months in function (20). The results showed that all crowns can survive a period equivalent to three months in the mouth. However, the total plastic framework could not survive when the use of temporary crowns was considered for a longer period.

The use of implant-supported temporary crowns usually seeks to associate aesthetic and minimal masticatory load (1-3,16) or even the absence of occlusal contacts (1). All these approaches aim to alleviate the mechanical problems that can occur if the temporary crown receives an excessive masticatory load. The present study demonstrated that not only high loads but also the mechanical fatigue can compromise the crown reliability.

The different framework types for definitive crowns are widely discussed in the literature, ranging from variations in geometry (21), material (22) and elastic modulus (18,23). However, there is a lack of data considering temporary crowns (11). It is also important to investigate temporary crowns, especially during the bone healing stage in the first 3 months after implant installation. This period is considered as the primary period for the clinical success to observe implant osseointegration (24). Therefore, the results of the present study confirm that the titanium framework is indicated to reduce early complications in this critical period because it presents a higher chance of survival.

A previous study (11) evaluated the effects of different frameworks on the stress distribution of implant-supported provisional PMMA single crowns. Was conclude that all evaluated framework types (Total plastic, Plastic with CoCr base and Titanium) can be used in the manufacturing of provisional crowns. However, with the results of the present study it is possible to limit the indication of plastic framework until 3 months of use while the other two frameworks can be used until 6 months.

In comparing the temporary crowns used on natural teeth, the implant-supported crowns presented a higher volume of acrylic resin. Still, its indication is restricted in short time periods due to the degradation that this material undergoes in the mouth because of the thermal cycling, pH and oral bacteria effects on this material. Therefore, the plastic framework enables the crown to have a 61% chance of survival following a 3-month protocol compared to 81% for the titanium framework. For the 6-month usage protocol under the simulated conditions, the totally plastic provisional crowns had 0% chance of survival. This is worrying because the use of a plastic framework to manufacture temporary crowns is commonly reported in the literature (1,13-15). Authors generally select plastic frameworks to improve gingival aesthetics due to the absence of metal in the emergence profile (1,14,15). Some authors (1,13) have reported that temporary crowns with plastic infrastructure did not present any masticatory or aesthetic problems after 6 months of use. Due to the different findings observed in this study, prospective clinical studies should be conducted to actually confirm the hypothesis that plastic frameworks present a higher prevalence of clinical problems than titanium frameworks for temporary crowns.

A systematic review and meta-analysis evaluating data from 1396 patients at baseline with a total of 2739 implants placed, reported that one of the most common technical complications was fractures in the temporary restorations (25). The authors concluded that immediately loaded implants demonstrated less crestal bone resorption during healing and a similar impact on peri-implant soft tissues, as well as advent of biological and technical complications when compared to delayed loaded implants. Thus, the use of an adequate framework for temporary crowns should reduce the number of technical complications associated with the benefits of the immediate loading protocol.

Some authors report that the success of dental implants is not only dependent on osseointegration, but also on the longevity of the temporary superstructures (26). These authors focused on studying the bond strength between Titanium framework and acrylic resin to reduce the debonding between these structures. All abutments used as frameworks in the present study presented grooves in their surfaces, and acrylic resin was carefully applied on the surface. All failures reported in this study occurred in the prosthetic screws.

**Figure 3:**
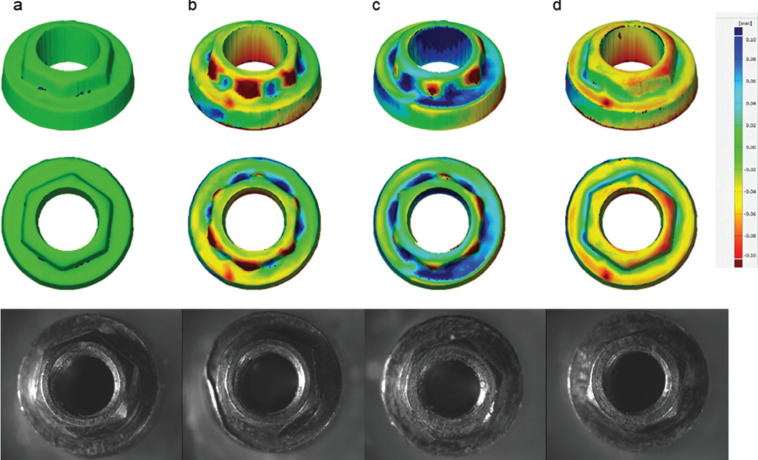
Implant platform 3D and microscopy a) prior to the fatigue test, fatigued implant with b) Titanium framework, c) Plastic framework with CoCr base and d) Total plastic framework.

In observing previous papers which performed dynamic fatigue in implants with external hexagon connection, it is possible to verify that damage in the prosthetic platform are commonly reported (27,28). The results of 3D implant overlapping before and after the fatigue corroborate these results, which can also be qualitatively observed in the photomicrographs in Figure 3. Therefore, the use of total Plastic framework did not damage the implant due to its flexibility and low hardness, which enabled a higher displacement of the crown during the load incidence and consequently higher damage to the prosthetic screw, justifying the lower survival of this group compared to the others.

The method used in this study to evaluate the damage in the implant platform was reported in studies that verified the effect of mechanical fatigue in dental ceramics (19) and dental enamel (29). The use of this analysis for dental implants had not yet been performed until now. Finally, it is important to note that the results of these *in vitro* investigations have limitations since all oral variations were not simulated (28) such as changes in pH, temperature, sliding occlusal loading, presence of bacteria, different antagonistic materials, and oblique forces (30). The aesthetics were also not evaluated (13). Even so, there is no information available in the literature on the fatigue of screw-retained temporary crowns. Therefore, this manuscript assists in justifying the use of Titanium frameworks for manufacturing temporary crowns.

Within the limitations of this study, it is possible to conclude that the framework type to manufacture implant-supported temporary crown influences the fatigue survival of the restoration. Moreover, the Plastic with CoCr base and Titanium framework are suitable for restorations over 3 months in use, without any difference in the implant platform damage.
